# An Account of Some Experiments Made with the Vapour of Boiling Tar, in the Cure of Pulmonary Consumption

**Published:** 1818-02

**Authors:** 


*An Account of some Experiments made with the Vapour of boiling Tar, in the Cure of Pulmonary Consumption.* By ALEXANDER CRICHTON, M.D. F.R.S. Physician in Ordinary to their Imperial Majesties the Emperor and Dowager Empress of Russia; Knight of the Order of St. Valdimir, &c. &c. 8vo. pp. 62. Edinburgh, 1818.

THIS is a very interesting tract from a respectable quarter on a very interesting subject, and we would be sorry that it should altogether escape the notice of our readers.—Phthisis has been justly styled the "climatorial disease" of Great Britain; and it is computed* that, by this fell scourge of youth and loveliness, not less than fifty thousand of our fellow subjects are annually swept away;—an extent of mortality sufficiently proving the headlong and hopeless nature of the disease.

We do not, indeed, go in with the opinion of M. Bayle, that every case of genuine consumption is absolutely incurable; seeing it is very apparent that medical treatment is often of avail to prolong, and sometimes to

---

* Dr. Johnson, on the Influence of the Atmosphere on Health, &c. Page 17.

save life: that the latter happy effect takes place, as we believe it does, once out of a hundred cases, is always something,—however short it may fall of the wishes of humanity, or however disproportionate it may be to the pretensions of an improving and extending science.

To lengthen out our remarks, however, on the tremendous and almost invariable fatality of this disease,—as the fact is so mournfully notorious—would be a vain expence of time; we apprehend, also, that many will conceive it equally a mis-spending of time to discuss accounts of new remedies which are every now and then proposed, and which, as usual, are big before-hand with the promise of success, but speedily betray that promise, and "break it to our hope," as soon as they are reduced to more accurate and more extensive trial.

We will not degrade our powers of reminiscence, by calling to mind the justly-forgotten farragos and nostrums of ancient or more modern times, which have, in their turn, been loudly extolled for the cure of phthisis: they and their inventors, pleased "to strut and fret their little hour," spontaneously went down "to the vile dust from whence they sprung." But we cannot help recollecting the brilliant proposal of a few years ago, associated as that recollection is with the affecting remembrance of departed genius, and the pride of high and genuine, though erratic, talent, now silenced in death! We allude to the genius and perseverance of the illustrious Beddoes, who first devised and extended the plausible aid of pneumatic medicine to the extenuated victims of pulmonary disease. All must remember, as we do, the brilliant hopes which his luminous suggestion excited in every quarter. Many a languid eye was re-lumed, and many a faded and anxious cheek took on a more cheerful expression, by this transient re-animation from despair! The delusive season, however, lasted not long; for certain it is, that the use of factitious airs disappointed both the patient and the physician. Whether it is that the effort to inhale them from bags, bladders, or gazometers, be too fatiguing, and therefore hurtful; or, whether their application be too temporary to effect benefit in any given case, we do not take upon us to say; but that they have not hitherto justified the previous hopes entertained of them, is beyond dispute.

It cannot be wondered at, then, that we should turn a somewhat incredulous ear to any new proposal, resting, as the *present* one undoubtedly does, on a *pneumatic* basis.

Our knowledge of the past has taught us the danger of hasty induction from a limited or premature experience. We always recollect the laconic advice, "*festina lentè,*" which, we think, might with propriety, on such occasions, be whispered in the ear of the ardent and the innovating. Yet, on the other hand, many *facts* lead us to believe that it is from the gazeous kingdom that we are most warranted to look for the means of alleviating or curing this disease, if ever, in the progress of our art to perfection, it should turn out to be more frequently susceptible of cure. These facts are, the injury which consumptive patients experience from the inhalation of oxygen, or even from breathing the air of dry and elevated situations; and the benefit they derive from residence in a contrary locality, viz. in low marshy districts, where the air obviously is impregnated with hydrogen, or with the hydro-carbonic gas. This has been well adverted to by our respected colleague, Dr. Johnson, at p. 22 of his new work on the Influence of the Atmosphere.

As bearing upon, and farther illustrating this point, we may be permitted to relate the following circumstance:—One individual of our CRITICAL TRIUMVIRATE has undoubted means of knowing, that, in his native country, intermittent fevers were remarkably rife about fifty or eighty years ago. At that time, consumption was a disease, comparatively speaking, known there only by hearsay. Of late years, however, owing to the gradual draining of the marshes and numerous low lands, together with many other agricultural improvements, a marked revolution has taken place in the Medico-Statistical History of the district: intermittents are quite extinct, and phthisis has now become as frequent and as fatal as elsewhere. Indeed, it was matter of popular remark, that consumptions had increased in the same ratio that agues had diminished. The real cause of this memorable change was, of course, too obscure to be appreciated by the vulgar; and, in consequence (as the above-mentioned individual, in his youth, well remembers), it was superstitiously attributed to the direct infliction of Providence, by the aged matrons of the time, whose mystical piety prompted them, at any rate, ever to soar above mere secondary causes, and to merge their less marvellous operation, in the more *taking* and " spirit-stirring" sublimity of supernatural agency. The circumstance was wont to be a frequent subject of melancholy animadversion amongst these sage persons at their set meetings and friendly compotations of *Bohea:* "The Lord hath taken away the ague, and in his anger, hath sent us the decline in its stead," was their remarkable expression; and it contained an awful idea which suddenly dashed, for a time, the pleasure from their *cups;*—moreover, being accompanied by a mournful shake of the head, it spread with infectious rapidity, and appalled the then infant mind of the Reviewer with sympathetic affright!

Now, though this description of persons, in their reasonings, are generally wrong, yet, in their relation of matters of fact, or of common observation, they are seldom mistaken; we therefore contend, that the foregoing, and many similar instances, prove, that an air in some degree vitiated with hydro-carbon, or, more properly speaking, with the oxy-carburetted hydrogen gas, is beneficial to pulmonic complaints; whereas, when this fluid is depurated by the natural physiognomy of a country, or by improvements wrought on its soil, in the same proportion it becomes irritant and unfavourable to tuberculated lungs.

If Dr. Crichton's plan shall be found to possess any utility, it will owe it entirely to this principle. Let us now, then, (as indeed it is high time) look more narrowly into the details of that plan.

Dr. C's object is, that the phthisical patient should breathe for several hours a-day, a factitious atmosphere, in many respects analogous to that which Nature furnishes in certain situations. For this purpose, he directs a small vessel, containing common tar (pix liquida, such as is commonly employed in the cordage of ships), to be placed over a spirit-lamp, taking care that it shall boil slowly, and not burn: thus the patient's room may be speedily filled with the vapour, and the fumigation be repeated every three hours. With each pound of tar Dr. C. mixes half an ounce of sub-carbonate of potass, in order to destroy what he calls the pyroligneous acid, but what we (with submission to an imperial physician) would call the empyreumatic acetic acid, which is generally found mixed with the tar, and excites cough.

The employment of this vapour, as is usual in such cases, was first suggested by accident. The author, from motives of curiosity or recreation, had gone one day, in the summer of 1816, to visit a cable manufactory in the vicinity of his summer residence. In the place where the tar was boiled, he found, to his great surprise, that though the vapour affected the eyes painfully, he breathed the air with perfect ease; it thence immediately struck him, that the air of the adjoining apartment, where the vapour and odour of tar were weaker, though strong, would form an artificial atmosphere that might be of use to a consumptive patient, who lived in the neighbourhood, and whom he had entirely despaired of curing. This patient's symptoms, so far as we can judge from the statement, were those of real phthisis: he had great oppression at the breast, purulent expectoration, and night sweats. Such was his state when he began the new process: the effects we shall relate in the Doctor's own words.

"The first day he remained in the tar-vapour four hours; and in a very short time after entering the room, he says he experienced a sensation of greater ease on the chest. As the weather was hot, and he had no one there to converse with, he fell asleep, but soon awoke with a head-ache; still the relief in his breathing was so decided, that he resolved to return to the room on the morrow. He continued daily to visit it, always falling asleep, and waking with head-ache. It was not till the expiration of some weeks that the tar-vapour ceased to have this effect. During this time, however, the cough and expectoration had gradually diminished; and at the end of a month, he had regained his former strength, when, without informing me, he went daily to town, neglected his remedy, and the cough returned. I reproached him for such imprudence, and insisted on his having recourse again to the tar vapour room, which had before been so successful. He returned to it, and continued his daily visits there until the end of our short summer. The beginning of September, he felt perfectly well, and thought his cure complete. I have since learned, in answer to a letter I addressed to him, that his health has continued good; that he has taken no medicine all the winter; and that he has gone out daily on business. P. 13, 14.

The unsuccessful result of this, and another similar case, encouraged Dr. C. to multiply the trials of the vapour. He therefore, by his influence with the court, procured it to be adopted in two of the civil hospitals of the city. He subjoins interesting Reports from the physicians of these establishments, detailing the favourable issue of the trial in eight cases, several of whom it seems to have cured, and the others to have materially relieved of strongly-marked phthisical symptoms. The average continuance of its use was from a month to six weeks; and the Reports add, that

"—it had a very quick and salutary effect on the cough, expectoration, and respiration; — that the sleep became more tranquil, continued, and restorative; and that the patients regained strength."

Thus, *ten* cases of its successful employment are narrated in this little tract; and if Dr. C. has not waited for a greater accumulation of evidence in favour of the remedy, it has arisen from a laudable desire to diffuse the knowledge of it as early as possible, lest any should perish unnecessarily, to whom the trial of this simple process might be salutary.

The author concludes his little work with the following observations:

"The patients who have derived the greatest benefit from the tar-vapour are those who were attacked with true scrophulous or tubercular phthisis. I confess this is quite contrary to what I expected. This kind of phthisis is the most common in Russia, as in all northern climates.

"The tar-vapour seems to have healed the ulcers, and removed the inflammation of the tubercles in the greater number of such cases; but I do not believe it produces the absorption of the tubercles themselves. My first patient, although well enough to attend his business out of doors; and, although he has regained his strength, has still some symptoms, which, to the eye of an observing physician, announce the presence of these tumours in his lungs; but so long as they remain inactive, that is to say, without inflammation or ulceration, life may be prolonged; and if circumstances allowed this patient to reside in a better climate, there is every probability that he might live long.

"In cases of phthisis derived from a large abscess or vomica, particularly in young persons of sanguineous constitutions, where suppuration goes on rapidly, and is generally accompanied by fever, the tar-vapour does little good. The immense quantity of purulent matter constantly present in the cavities of these large abscesses, prevents the vapour from acting on their surface, and consequently it loses its effect. In two cases of this kind, a very temporary relief only took place from the fumigation.

"Nearly the same thing may be said of cases of suppuration succeeding active hæmorrhage in young persons, especially when accompanied with fever.

"In one case, however, of this kind, I succeeded in removing the consumptive symptoms by the tar fumigation and the use of acetate of lead, combined with the aqueous extract of opium, which the patient continued to take during a whole month, without experiencing colic, or any other inconvenience.

"At that period, when the cough, expectoration, and hectic fever, are greatly subdued by the influence of the tar fumigation, it seems to me often injudicious to continue it longer, or at least in so strong a degree as before; it then appears to dry the lungs too much, and ends in exciting a spasmodic kind of cough. The patient, also, when in a state to breathe the common air, becomes weakened by the want of it. In a remarkable case of tubercular phthisis, where the tar-vapour had produced extraordinary benefit, I was obliged to suspend, every now and then, the use of it, particularly when the expectoration was very slight. During these suspensions, however, which never exceeded two or three days, I always found the expectoration, and other bad symptoms, increase." P. 58—60.

The following sentence, with which we shall conclude our extracts, shews that our author has nothing the most distant of the empiric about him, if ever that could have been supposed :

"Notwithstanding the great power of this means of cure, I never employed it quite alone, but have, at the same time, prescribed internal remedies, such as the nature and urgency of the symptoms seemed to require; but these have been the same as every practical physician has recourse to in similar cases." P. 61.

We have now given a full and fair view of the whole contents of this tract: the nature of the subject demanded it of us; and if our readers have sensibilities akin to ours, they will read these contents with no ordinary degree of interest. Upon the whole, distrustful as we are, and have reason to be, of new remedies in phthisis, we would greatly like to see Dr. Crichton's plan tried in this country, inasmuch as we think there is at least sufficient evidence of its being quite harmless in chronic pectoral complaints. Besides, the ill success of the usual means authorizes us to embrace a remedy proposed by so respectable an individual, even should it be productive of less benefit than the proposer alleges. In giving it a fair trial, we ought to remember the words of Dr. Young, that—

"—we must have witnessed the failure of any new mode of treatment, in at least fifty cases, before we are fully authorized to suppose that it has been less successful than the most effectual remedies previously known." *

While upon the subject of novel treatment in pulmonary consumption, it may not be altogether irrelevant to mention the leading features of a plan first proposed, and pursued of late, by A. Stewart, M. D. a Scottish clergyman. It was in high repute some years ago, and indeed is so still, amongst many of the higher classes in that part of the kingdom; as many of our readers have probably never heard of it, we shall briefly state its outlines.

This gentleman's postulatum, then, is, that consump-

* See Dr. Young's Practical and Historical Treatise on Consumptive Diseases, p. 52.

tion is scrophula; and he holds, that the same principle ought to guide the medical treatment in the former as in the latter. He, therefore, comparatively overlooks the idea of inflammation, except in the very first stage, and chiefly aims at reproducing, what he calls, " a cool, braced, and seasoned state of the constitution." With this view, he prohibits an aqueous or vegetable diet, and orders the patient to satisfy his appetite with food that is nutritious, though not stimulating: such as eggs, fresh animal food, veal broths, jellies, and arrow-root. For drink he prescribes Port wine and water in small quantities, water acidulated with sulphuric acid, and spruce, ginger, or small beer. Abundance of gestation-exercise, both without doors and within, is also enjoined; and the whole surface of the body is sponged daily with water and vinegar, at first tepid, but gradually cooled down to the temperature of the air. This is to be followed up by friction with hard dry cloths, or with the flesh-brush.

As to medical means, properly so called, Dr. Stewart uses only bark-infusion with sulphuric acid twice a day, or a preparation of steel with a bitter, and a blister-issue on the breast.

Several cures of patients, particularly females, in high life, attest the superiority of this plan: but, that it is generally by no means admissible, we are quite satisfied: and, indeed, of those said to be cured by it, it may fairly be questioned whether any actually laboured under real phthisis, or merely under some cachectic or anomalous complaints, simulating (as we know they frequently do) the phænomena and progress of genuine pulmonary consumption. This doubt has been entertained by several eminent physicians on the spot, who are well qualified to form a correct judgment on such matters.

